# Role of Horizontal Gene Transfer in the Development of Multidrug Resistance in Haemophilus influenzae

**DOI:** 10.1128/mSphere.00969-19

**Published:** 2020-01-29

**Authors:** Kristin Hegstad, Haima Mylvaganam, Jessin Janice, Ellen Josefsen, Audun Sivertsen, Dagfinn Skaare

**Affiliations:** a Norwegian National Advisory Unit on Detection of Antimicrobial Resistance, Department of Microbiology and Infection Control, University Hospital of North-Norway, Tromsø, Norway; b Research Group for Host-Microbe Interactions, Department of Medical Biology, Faculty of Health Sciences, University of Tromsø (UiT)—The Arctic University of Norway, Tromsø, Norway; c Department of Microbiology, Haukeland University Hospital, Bergen, Norway; d Department of Microbiology, Vestfold Hospital Trust, Tønsberg, Norway; University of Nebraska Medical Center

**Keywords:** *Haemophilus influenzae*, integrative conjugative element, multidrug resistant, horizontal gene transfer, PBP3, beta-lactam resistance, mobile genetic element

## Abstract

Haemophilus influenzae colonizes the respiratory tract in humans and causes both invasive and noninvasive infections. Resistance to extended-spectrum cephalosporins in H. influenzae is rare in Europe. In this study, we defined acquired resistance gene loci and *ftsI* mutations in multidrug-resistant (MDR) and/or PBP3-mediated beta-lactam-resistant (rPBP3) H. influenzae strains, intending to understand the mode of spread of antibiotic resistance determinants in this species. Horizontal transfer of mobile genetic elements and transformation with resistance-conferring *ftsI* alleles were contributory. We found one small plasmid and three novel integrative conjugative elements (ICEs) which carry different combinations of resistance genes. Demonstration of transfer and/or ICE circular forms showed that the ICEs are functional. Two extensively MDR genetically unrelated H. influenzae strains (F and G) from the same geographical region shared an identical novel MDR ICE (Tn*6686*) harboring *bla*_TEM-1_, *catA2*-like, and *tet*(B). The first Nordic case of MDR H. influenzae septicemia, strain 0, originating from the same geographical area as these strains, had a similar resistance pattern but contained another ICE [Tn*6687* with *bla*_TEM-1_, *catP* and *tet*(B)] with an overall structure quite similar to that of Tn*6686.* Comparison of the complete *ftsI* genes among rPBP3 strains revealed that the entire gene or certain regions of it are identical in genetically unrelated strains, indicating horizontal gene transfer. Our findings illustrate that H. influenzae is capable of acquiring resistance against a wide range of commonly used antibiotics through horizontal gene transfer, in terms of conjugative transfer of ICEs and transformation of chromosomal genes.

**IMPORTANCE** Haemophilus influenzae colonizes the respiratory tract in humans and causes both invasive and noninvasive infections. As a threat to treatment, resistance against critically important antibiotics is on the rise in H. influenzae. Identifying mechanisms for horizontal acquisition of resistance genes is important to understand how multidrug resistance develops. The present study explores the antimicrobial resistance genes and their context in beta-lactam-resistant H. influenzae with coresistance to up to four non-beta-lactam groups. The results reveal that this organism is capable of acquiring resistance to a wide range of commonly used antibiotics through conjugative transfer of mobile genetic elements and transformation of chromosomal genes, resulting in mosaic genes with a broader resistance spectrum. Strains with chromosomally mediated resistance to extended-spectrum cephalosporins, co-trimoxazole, and quinolones combined with mobile genetic elements carrying genes mediating resistance to ampicillin, tetracyclines, and chloramphenicol have been reported, and further dissemination of such strains represents a particular concern.

## Abstract (precis)

Haemophilus influenzae colonizes the respiratory tract in humans and causes both invasive and noninvasive infections. As a threat to treatment, resistance against critically important antibiotics is on the rise in H. influenzae. Identifying mechanisms for horizontal acquisition of resistance genes is important to understand how multidrug resistance develops. The present study explores the antimicrobial resistance genes and their context in beta-lactam-resistant H. influenzae with coresistance to up to four non-beta-lactam groups. The results reveal that this organism is capable of acquiring resistance to a wide range of commonly used antibiotics through conjugative transfer of mobile genetic elements and transformation of chromosomal genes, resulting in mosaic genes with a broader resistance spectrum. Strains with chromosomally mediated resistance to extended-spectrum cephalosporins, co-trimoxazole, and quinolones combined with mobile genetic elements carrying genes mediating resistance to ampicillin, tetracyclines, and chloramphenicol have been reported, and further dissemination of such strains represents a particular concern.

## INTRODUCTION

Haemophilus influenzae colonizes the respiratory tract in humans and causes both invasive and noninvasive infections. The most common type of H. influenzae that caused invasive infections used to be encapsulated strains that belonged to serotype b (Hib). After the commencement of vaccination against Hib, the overall incidence of invasive infections has decreased considerably, and invasive infections due to other serotypes (a and f) and noncapsulated strains (nontypeable; NTHi) have emerged (1).

As a threat to treatment, resistance against critically important antibiotics is on the rise in H. influenzae (2, 3). Beta-lactamase-producing strains emerged and increased rapidly in the 1970s, effectively eliminating traditional first-choice beta-lactamase-susceptible penicillins as safe options for empirical therapy. The most common horizontally acquired beta-lactamase gene in H. influenzae is *bla*_TEM-1_, originating from Escherichia coli (4). Acquisition of genes encoding extended-spectrum beta-lactamases (ESBLs) is reported for Haemophilus parainfluenzae (*bla*_TEM-15_) (5) but not yet for H. influenzae.

Horizontal gene transfer (HGT) of *bla*_TEM-1_ in H. influenzae is facilitated by small nonconjugative plasmids in a small proportion of isolates (6, 7), but the gene is more frequently transferred by integrative conjugative elements (ICEs) (8). In addition to *bla*_TEM-1_, ICEs may carry genes mediating resistance to chloramphenicol (*cat*, encoding chloramphenicol acetyltransferase) (9) and tetracyclines [*tet*(B), encoding efflux of tetracycline from the bacterial cell] (10). The first Nordic case of septicemia caused by a multidrug-resistant (MDR) H. influenzae with this phenotype was reported in 1983 (11).

ICEs are major effectors in genome dynamics in bacteria, being the most abundant conjugative elements in prokaryotes (12). They are self-transmissible mobile genetic elements (MGEs) that encode an apparatus for their own excision from the donor chromosome, subsequent circularization, conjugation, and reintegration into the recipient chromosome (13). ICEs often contain site-specific integrases which help the ICE to recombine at specific attachment (*att*) sites (14).

Genes involved in excision/integration and conjugation are clustered into distinct modules in the ICEs. In H. influenzae, ICEs harbor both an excisase and an integrase in their integration module, and their conjugation module consists of a conserved cluster of 24 genes constituting a particular lineage of type IV secretion systems (T4SSs) (15). The T4SS gene cluster encodes both proteins involved in DNA processing and transfer (relaxase and coupling protein being the two main parts) and mating pair formation (secretion channel, motor proteins, and retraction pilus/surface adhesin) (16).

Since 2000, chromosomal mechanisms conferring resistance to other classes of beta-lactams have become widespread in H. influenzae (2). The most important mechanism of non-beta-lactamase-mediated beta-lactam resistance in H. influenzae is altered penicillin-binding protein 3 (PBP3), which develops through spontaneous point mutations and/or transformation within the transpeptidase region of the *ftsI* gene (17). Resistance to extended-spectrum cephalosporins (ESC-R) is endemic in Japan and Korea (1). The ESC-R phenotype appears to develop stepwise along two distinct pathways, where a first stage substitution (N526K or R517H) is required for the acquisition of second stage (S385T) and third stage (L389F) substitutions (17, 18). This has given rise to a classification system for PBP3-mediated beta-lactam resistance (rPBP3) with six distinct resistance genotypes, denoted groups I, II, III, III-like, III+, and III-like+ (17, 19, 20).

There is strong evidence that resistance-conferring *ftsI* alleles could arise *in vivo*, either spontaneously or horizontally acquired by transformation. Several *in vitro* experiments have shown that complete or partial *ftsI* genes may be used to transform susceptible strains (17, 18, 21–23), and identical mutated *ftsI* alleles have been reported in genetically unrelated clinical strains (20, 24, 25). H. influenzae is naturally competent, with the ability to transform through DNA uptake and homologous recombination (26). Recognition and efficient DNA uptake depend on the presence of specific 9-bp uptake signal sequences (USS) in the donor molecule, with a 4-bp core being particularly important (27).

Transformation may also contribute to resistance to co-trimoxazole and quinolones, but the relative importance of this mechanism compared to that of spontaneous point mutations is, to our knowledge, incompletely investigated in H. influenzae. Resistance to co-trimoxazole is caused by reduced activity of trimethoprim due to alterations in the *dfrA* gene encoding dihydrofolate reductase (28) and/or resistance to sulfamethoxazole due to acquisition of *sul* genes or mutations in the *folP* gene encoding dihydropteroate synthase (29). Resistance to quinolones is usually due to substitutions in the quinolone resistance-determining regions of topoisomerase II (GyrA) and IV (ParC) (30). Whereas co-trimoxazole resistance is common, resistance to quinolones is still infrequent in most parts of the world. A notable exception is the emergence of levofloxacin-resistant H. influenzae clones in Taiwan (31).

Importantly, resistance to multiple classes of antibiotics may occur in the same strain through a combination of MGEs and chromosomal mechanisms, exemplified by the clonal expansion of ESC-R strains with *bla*_TEM-1_ and coresistance to ciprofloxacin, tetracycline, chloramphenicol, and co-trimoxazole in Norway (20).

In this study, we defined acquired resistance gene loci and *ftsI* mutations in Norwegian MDR and/or rPBP3 H. influenzae strains, with the aim to explore the mode of spread of antibiotic resistance determinants in this species through horizontal transfer of MGEs and transformation with resistance-conferring *ftsI* alleles.

## RESULTS AND DISCUSSION

Analyses of the whole-genome sequencing (WGS) data confirmed that seven of the isolates in our collection had the acquired *bla*_TEM-1_ beta-lactam resistance gene, and five of these isolates had also acquired resistance genes to chloramphenicol (*catA*-like, *catP*) and tetracycline [*tet*(B)], which matched their phenotypic profiles (Table 1).

**TABLE 1:** Isolates and their characteristics^*a*^

Strain or isolate	Reference	Yr of isolation	Site of origin	Phenotypic resistance^*b*^		WGS clade^*c*^	MLST^*d*^		Serotype^*e*^	PBP3 resistance			*ftsI* type^*h*^	Acquired resistance gene(s)^*i*^	Mobile genetic element
				Beta-lactam(s)	Other agent(s)		CC	ST		Level^*f*^	Group^*f*^	Type^*g*^			
0	11	1983	Blood	A	Te, Ch	VI	6	119	Hib	sPBP3	sPBP3	z	*alpha-x*	*bla*_TEM-1_, *catP*, *tet*(B)	Tn*6687*
A	24	2007	Ear	Cf	Tx	IV	57	57	NT	Low	II	A	*lambda-2*	—	—
B	24	2007	Npx^*j*^	Cf	—	V	3	367	NT	Low	II	A	*lambda-2*	—	—
C	24	2007	Ear	Cf	—	V	12	12	NT	Low	II	H	*gamma*	—	—
D	24	2007	Npx	Cf	—	IV	422	411	NT	Low	II	H	*gamma*	—	—
E	20	2010	Eye	A, Ac, Cf, Ct, Cx	Tx, Ci	IV	422	422	NT	High	III-like+	3	*ftsI-4*	*bla*_TEM-1_	Tn*6685*
F	20	2013	Sputum	A, Ac, Cf, Ct, Cx	Tx, Te, Ch	II	503	1282	NT	High	III-like+	3	*ftsI-4*	*bla*_TEM-1_, *catA*-like, *tet*(B)	Tn*6686*
G	20	2013	Sputum	A, Ac, Cf, Ct, Cx	Tx, Te, Ch, Ci	II	503	159	NT	High	III+	2	*ftsI-5*	*bla*_TEM-1_, *catA*-like, *tet*(B)	Tn*6686*
G2	20	2013	Eye	A, Ac, Cf, Ct, Cx	Tx, Te, Ch, Ci	II	503	159	NT	High	III+	2	*ftsI-5*	*bla*_TEM-1_, *catA*-like, *tet*(B)	Tn*6686*
G3	20	2013	Npx	A, Ac, Cf, Ct, Cx	Tx, Te, Ch, Ci	II	503	159	NT	High	III+	2	*ftsI-5*	*bla*_TEM-1_, *catA*-like, *tet*(B)	Tn*6686*
H	20	2013	Npx	A, Ac, Cf, Ct, Cx	Tx	V	245	836	NT	High	III+	2	*ftsI-2*	*bla*_TEM-1_	pH*bla*_TEM-1_
I	20	2012	Ear	A, Ac, Cf, Ct, Cx	—	I	124	124	Hif	High	III+	2	*ftsI-2*	—	—

a Shading indicates identical resistance-conferring *ftsI* alleles or MGE shared by different strains or isolates.

b A, ampicillin; Ac, amoxicillin-clavulanic acid; Cf, cefuroxime; Ct, cefotaxime; Cx, ceftriaxone; Tx, co-trimoxazole; Te, tetracycline; Ch, chloramphenicol; Ci, ciprofloxacin; —, none. For MICs and clinical breakpoints, see Table S2 in the supplemental material.

c Whole-genome phylogeny with assignment to phylogenetic groups according to De Chiara et al. (50).

d MLST, multilocus sequence typing; CC, clonal complex (named after predicted founder by eBURST analysis); ST, sequence type.

e Hib, serotype b; Hif, serotype f; NT, nontypeable.

f Based on amino acid substitutions in penicillin-binding protein 3 (PBP3), positions 385, 389, 517, and 526. sPBP3, no substitutions; Low, N526K or R517H; High, S385T in addition to N526K or R517H; II, N526K; III-like+, S385T, L389F, R517H; III+, S385T, L389F, N526K (20, 24).

g Based on amino acid sequences in positions 350, 357, 377, 385, 389, 502, 517, 526, 532, 547, 557, 562, and 569 (substitutions underlined): z, DSMSLARNTVYVN (identical to the reference sequence Rd KW20 [53]); A, NSISLVRKTIYVS; H, DSMSLVRKTVYVN; 3, NNITFAHNSIHVS; 2, NNITFARKTIYLS (20, 24).

h Based on partial nucleotide sequences in the transpeptidase domain of the *ftsI* gene (nt 1010 to 1719) (20, 24). The *ftsI* type for strain 0 clusters with Rd KW20 in group *alpha* (24) and is therefore assigned *alpha-x*.

i *bla*, beta-lactamase gene; *catA* and *catP*, chloramphenicol resistance genes; *tet*(B), tetracycline resistance gene; —, none.

j Npx, nasopharynx.

ICEs and small plasmids have been described to house acquired resistance genes and mediate horizontal transfer of these genes in H. influenzae. Therefore, we looked more closely at the contigs containing the acquired resistance genes.

### Strain H: plasmid with *bla*_TEM-1_.

In strain H, the *bla*_TEM-1_ gene was shown to be part of a contig with homology to small plasmids in H. influenzae and Haemophilus parainfluenzae (data not shown). Gap closure of the *bla*_TEM-1_ contig showed that the *bla*_TEM-1_ plasmid in strain H is identical to the small 5,142-bp H. influenzae plasmid pA1209 (data not shown), isolated from a patient in the Aarhus region in Denmark (6). This plasmid contained three open reading frames (ORFs): (i) an ORF encoding a replication protein of the Rep 3 superfamily typical of small resistance plasmids in *Pasteurellaceae*, (ii) the *bla*_TEM-1_ gene, and (iii) an ORF with partial identity to a plasmid recombination enzyme that may help the plasmid recombine into other replicons and thereby get mobilized. Mating was attempted using strain Rd-Rif as a recipient, but no transconjugants were found within the detection limit (<1 × 10^−11^ transconjugants/donor). This is not surprising, since strain H does not harbor all the genes necessary for conjugation, as determined by running the genome in the web-based bacterial type IV secretion system resource, SecReT4 (32).

### Strain E: novel ICE (Tn*6685*) with *bla*_TEM-1_.

**FIG 1:**
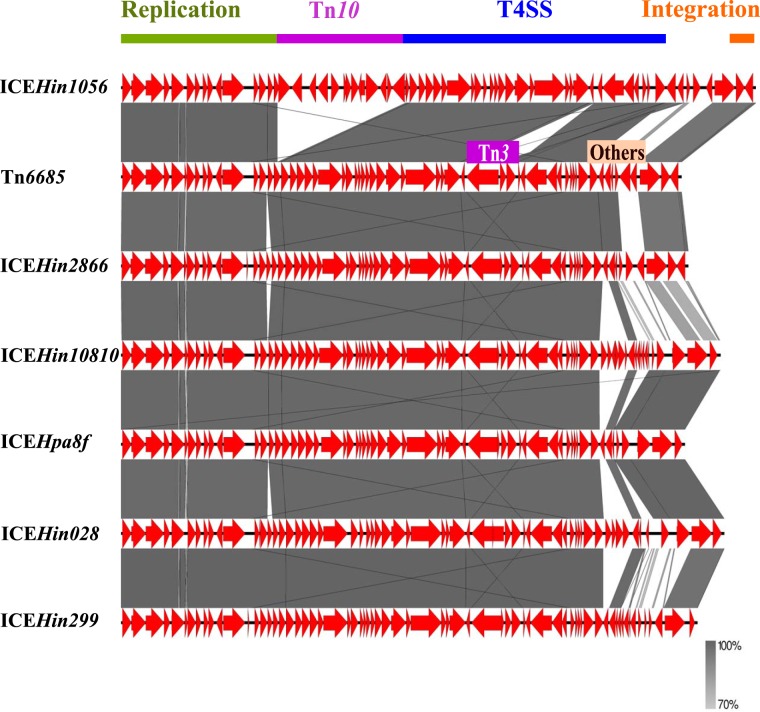
Pairwise comparison of the *bla*_TEM-1_-containing ICE of strain E (Tn*6685*) with representative ICEs containing *bla*_TEM-1_ previously described in H. influenzae. Transposable elements as well as functional regions are indicated. The gray bands represent the forward matches. In contrast to Tn*6685*, ICE*Hin1056* contains Tn*10* which harbors *cat* and *tet* resistance genes but has a nonfunctional Tn*3* which harbors *bla*_TEM-1_ and lacks the transposase. T4SS, type IV secretion system conjugation module.

The *bla*_TEM-1_ gene in strain E was located within a novel 52.4-kb ICE (Tn*6685*) which is most similar to the previously described 53.0-kb ICE*Hin2866* (33) (Fig. 1). Tn*6685* contains three ORFs that are not present in ICE*Hin2866*. These encode a hypothetical protein and two products with conserved domains, indicating they belong to the type IV toxin-antitoxin system, part of the nucleotidyltransferase AbiEii superfamily. The AbiEii toxin and its cognate transcriptional regulator AbiEi antitoxin belong to abortive bacterial infection (Abi) systems. These can have a dual function, namely, (i) protecting bacteria from spread of a phage infection by aborting the cell upon phage infection, and (ii) ensuring stability of MGEs containing such toxin-antitoxin systems by killing cells that lose the MGE upon cell division (34).

### Strains F and G: novel ICE (Tn*6686*) with *bla*_TEM-1_, *catA2*-like, and *tet*(B).

Strain F and three isolates of strain G (G, G2, and G3), isolated within a time span of 2 weeks from four patients living in the same geographical region (20) and belonging to the same clonal complex (CC-ST503), harbored identical acquired resistance genes against penicillins (*bla*_TEM-1_), chloramphenicol (*catA*-like; 90% identity to *catA2* [GenBank accession number X53796]), and tetracycline [*tet*(B)]. Isolates G, G2, and G3 had the same multilocus sequence type (MLST) and phenotypic resistance pattern and identical *ftsI* gene, indicating they belong to the same strain. Core genome phylogeny supports that isolates G, G2, and G3 are very closely related: G has 13 and 12 single nucleotide polymorphisms (SNPs) in the core genome compared to G2 and G3, respectively, whereas G2 and G3 have only a two-SNP difference between them. Strain F showed a related ST with six of seven MLST alleles in common with strain G. However, strain F is not closely related to strain G, since it has 1,493 core SNP differences compared to strain G, a slightly different resistance pattern and a different PBP3 type than strain G.

**FIG 2:**
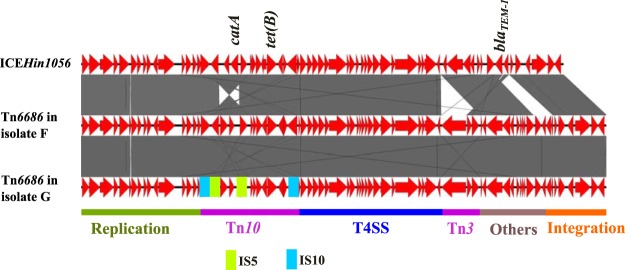
Pairwise comparisons of the novel ICE Tn*6686* from strains F and G with ICE*Hin1056*. Insertion sequences and other transposable elements as well as functional regions are indicated. In contrast to ICEHin*1056*, the novel ICE contains a functional Tn*3* which harbors *bla*_TEM-1_. Other resistance genes are also indicated. The gray bands represent the forward and reverse matches.

**FIG 3:**
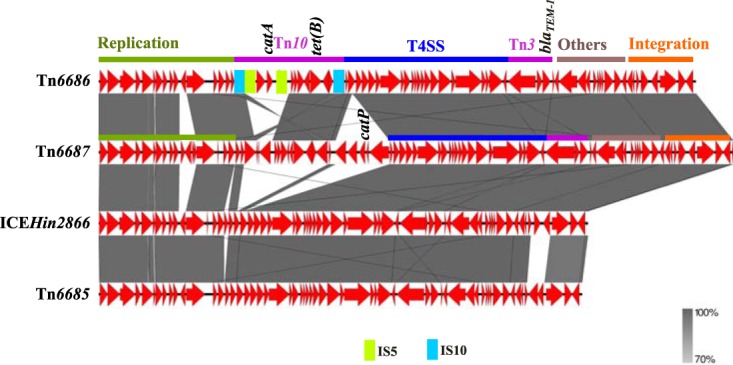
Pairwise comparisons of the novel ICE Tn*6687* from strain 0, the multidrug-resistant H. Influenzae isolate from 1983, with Tn*6686*, Tn*6685*, and ICE*Hin2866* representing ICEs containing *bla*_TEM-1_ previously described in H. influenzae. Insertion sequences and other transposable elements as well as functional regions are indicated. Resistance genes are indicated. The gray bands represent the forward and reverse matches.

Pairwise BLAST revealed the presence of large fragments of ICE*Hin1056* (GenBank accession number [acc. no.] AJ627386) (35) in all four isolates. This ICE encodes resistance to penicillins, chloramphenicol, and tetracycline. Gap closures revealed a 100% identical putative novel 64.7-kb ICE (Tn*6686*) in all four isolates belonging to strain F and strain G. The presence of an identical ICE in the two different strains as well as close epidemiological connection between strain F and G indicate that transfer of this ICE has occurred between these strains. The overall structure of Tn*6686* is quite similar to that of ICE*Hin1056* (Fig. 2). This novel ICE has an inverted region around the *catA*-like gene due to IS*5* elements and a complete Tn*3* element, including the transposase gene that is missing in ICE*Hin1056*. Tn*6686* has some additional ORFs not present in ICE*Hin1056*, including ORFs encoding a site-specific recombinase/DNA invertase homologue that is found in other ICEs of *Haemophilus* such as ICE*Hin2866* (Fig. 3) and Actinobacillus pleuropneumoniae ICE*Apl1*, and a type I restriction modification system subunit M with 100% identity to methyltransferases found in other ICEs of *Haemophilus* (GenBank acc. no. ADO80528.1) and A. pleuropneumoniae (GenBank acc. no. ANC65583.1). However, the other subunits necessary for restriction modification function (36) are missing.

### Strain 0: novel ICE (Tn*6687*) with *bla*_TEM-1_, *catP* and *tet*(B).

Strain 0 caused the first Nordic case of MDR H. influenzae septicemia (11) and originates from the same geographical area as strains F and G. As strains 0, F, and G have similar resistance patterns, except for the lack of ESC-R in strain 0 (sensitive PBP3 genotype/phenotype [sPBP3]) (Table 1), we were curious whether the much older strain 0 contains the same ICE as the novel Tn*6686* found in strains F and G. However, the acquired resistance genes in strain 0 turned out to be different, as this strain contains *catP* rather than the *catA*-like gene found in strains F and G. Gap closure revealed that strain 0 contains a novel 68.8-kb ICE (Tn*6687*) containing *bla*_TEM-1_, *catP*, and *tet*(B). Comparisons of Tn*6687* with Tn*6685*, Tn*6686*, and ICE*Hin2866* showed that Tn*6687* is more similar to Tn*6686* than to other ICEs previously described in H. influenzae (Fig. 2 and 3). The overall structure is also quite similar to Tn*6686* except for some additional putative ORFs and that the *cat* gene regions are different and on opposite sides of the *tet*(B) region. The *catP* gene is surrounded by putative ORFs encoding a hypothetical protein with similarity to plasmid recombination proteins, an AAA family ATPase similar to the uncharacterized protein TnpY encoded by the mobilizable transposon Tn*4451*, and a TndX-like transposase, suggesting it is part of a smaller MGE. Moreover, Tn*6687* contains a *gltS* gene encoding a sodium-glutamate symporter important for growth with glutamate as carbon and nitrogen sources (37).

### Integration and transferability of novel ICEs.

ICEs are expected to circularize after excision (13). A circular form of the ICE was demonstrated in all isolates containing novel ICEs (strains E, F, G/G2/G3, and 0) by PCRs using primers directed outwards from the integrated ICE (see Fig. S1 in the supplemental material). Sequencing of the PCR products confirmed the expected joined right and left end sequences and determination of the correct ends of the ICEs (data not shown). These results demonstrate that the enzymes involved in excision of the ICEs are functional and indicate that the ICEs are potentially transferable.

10.1128/mSphere.00969-19.1 FIG S1 Schematic drawing of integrated and circular forms of the ICEs (Tn*6685*, Tn*6686*, and Tn*6687*). Left and right ICE ends are colored gray and black. Native chromosomal DNA is shown in red. Primer positions for demonstration of ICE circular forms are indicated. Download FIG S1, PPTX file, 0.1 MB. Copyright © 2020 Hegstad et al. 2020 Hegstad et al. This content is distributed under the terms of the Creative Commons Attribution 4.0 International license.

All the novel ICEs have the highly conserved cluster of genes encoding T4SS products mediating conjugation (Fig. 1 and 3). Mating was attempted using strains E, F, G, and 0 as donors. ICE*Hin1056* transfer frequency was previously shown to range from 10^−1^ to 10^−9^ (33). Transconjugants were obtained and confirmed with strains F, G, and 0 as donors (transfer frequencies ranging from 5 × 10^−7^ to 6 × 10^−9^ transconjugants/donor), confirming that the novel ICEs in these strains are transferable. Transconjugants containing Tn*6685* using strain E as donor were not obtainable.

**FIG 4:**
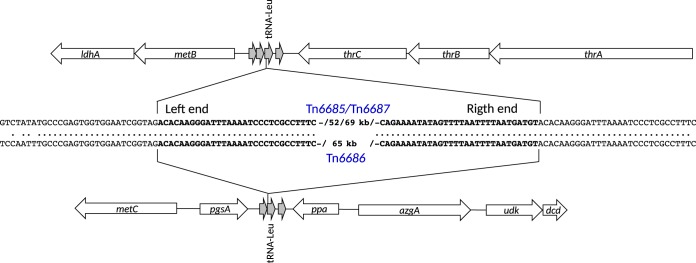
Schematic drawing of the integration site of novel ICEs with an enhanced focus on the nucleotides in the flanking regions in the middle of the figure. Gray arrows represent tRNAs. Bold letters represent ICE ends. Dots represent homology between nucleotides. Repeats are in bold. Only 30 of the 66 nucleotides that perfectly directly repeat in the insertion region are shown in the figure.

Comparison of the integration sites and right and left ends of all the ICEs, including ICE*Hin1056*, shows that the ICE ends and part of the integration sites are identical; thus, these ICEs integrate in a site-specific manner. All the ICEs are inserted downstream of the 22nd nucleotide in tRNA-Leu. The integration site tRNA-Leu appears in two copies in H. influenzae, in separate chromosomal locations. This explains why the chromosomal location of Tn*6685* and Tn*6687* is different from that of Tn*6686* (Fig. 4).

### HGT of resistance-conferring *ftsI* alleles.

**FIG 5:**
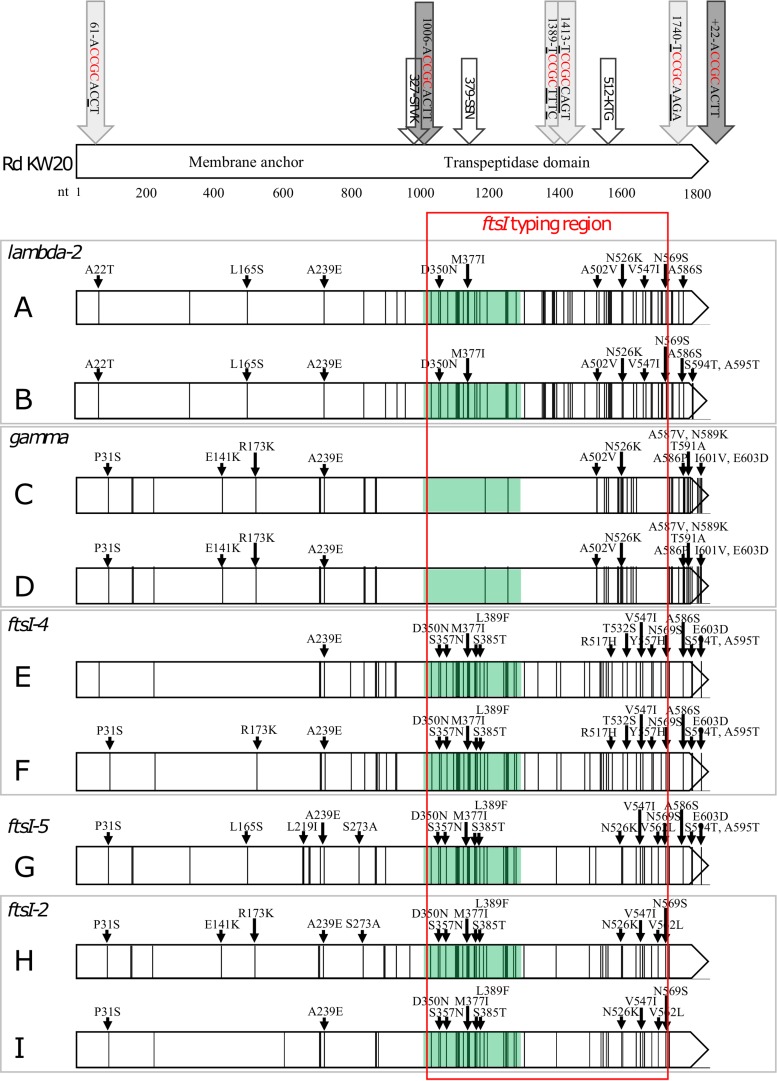
DNA mutations and amino acid substitutions in the *ftsI* gene (nt 1 to 1833) of strains A to I compared to Rd KW20. Isolates G2 and G3 share identical *ftsI* genes with strain G and are therefore not included in the figure. Each vertical line represents a SNP. Black small arrows point to where amino acid differences occur. The light gray arrows pointing at the Rd KW20 gene represent USS variants with minor mismatches compared to the USS consensus sequence (dark gray arrows) (27). White arrows indicate the conserved STVK, SSN, and KTG motifs. The *ftsI* typing region (nt 1010 to 1719) and SSN-near region (nt 1006 to 1295) are shown by red frame and green shading, respectively. Strains with identical *ftsI* types are framed in gray. For a more detailed presentation of the SSN-near region, see Table S1 and Fig. S2 to S4 in the supplemental material.

Figure 5 shows the distribution of SNPs and substitutions in *ftsI*/PBP3 of strains A to I.

10.1128/mSphere.00969-19.2 FIG S2 Phylogenetic tree showing the relative difference between the SSN-near region (nt 1006 to 1295) of the *ftsI* gene in the *ftsI* types represented in the present study (Table 1) and the reference sequence Rd KW20 (53). Text color indicates resistance genotype: green, sPBP3; blue, low-rPBP3; red, high-rPBP3. The phylogenetic tree was constructed using the neighbor-joining method implemented in Neighbor from the PHYLIP package (v3.696) and visualized with TreeDyn (v198.3); the software is available online at http://www.phylogeny.fr. Download FIG S2, PDF file, 0.1 MB. Copyright © 2020 Hegstad et al. 2020 Hegstad et al. This content is distributed under the terms of the Creative Commons Attribution 4.0 International license.

10.1128/mSphere.00969-19.5 TABLE S1 Comparison of the SSN-near region (nt 1006 to 1295) of the *ftsI* gene in the *ftsI* types represented in the present study (Table 1). Download Table S1, DOC file, 0.1 MB. Copyright © 2020 Hegstad et al. 2020 Hegstad et al. This content is distributed under the terms of the Creative Commons Attribution 4.0 International license.

As reported previously (24), the genetically unrelated strains within each of the strain pairs A/B and C/D share identical *ftsI* alleles (*lambda-2* and *gamma*, respectively). These strains, collected in 2007, are low-level resistant (group II rPBP3) and susceptible to ESC (Table 1). Comparison of complete *ftsI* genes revealed that the genes are identical in strains A and B, except for three mutations in the 3′ end of the gene in strain B leading to two additional amino acid substitutions (S594T and A595T), whereas strains C and D share completely identical *ftsI* genes (Fig. 5). Similarly, the genetically unrelated strains within each of the strain pairs E/F (group III-like+) and H/I (group III+) isolated between 2010 and 2013, all categorized as high rPBP3 and expressing the ESC-R phenotype, share identical *ftsI* types (*ftsI-4* and *ftsI-2*, respectively), despite being genetically unrelated, as depicted by WGS and MLST phylogeny (Table 1) (20). However, the nucleotide sequences upstream of the STVK motif in the strain pairs E/F and H/I differ at 3 and 13 positions, respectively, indicating possible HGT with recombination affecting only the transpeptidase region of *ftsI*.

As shown in Fig. 5, a fragment of the transpeptidase region surrounding the SSN motif (green shading) was identical or highly similar in strains E, F, H, and I, as well as in strain G, which belongs to the same clonal complex as strain F but has a different MLST profile. Alignment of the SSN-near region (nucleotide [nt] 1006 to 1295) showed that the *ftsI* types carried by strains E to I (*ftsI*-2, *ftsI*-4, and *ftsI*-5) had a maximum one-SNP difference between them, while they differed from the reference sequence by 24 to 25 SNPs (<92% identity) (Table S1 and Fig. S2 and S3). This observation is of particular significance, as this region harbors the second- and third stage-substitutions S385T and L389F, with the potential to transform low-rPBP3 strains into high-rPBP3 strains (group III-like+ or III+), which in most cases will express resistance to ESC. Notably, *ftsI*-2, *ftsI*-4, or *ftsI*-5 was present in 12 of 16 isolates with high+ (group III-like+ or III+) genotypes in a previous report on the first 30 high-rPBP3 strains reported in Norway (20).

These findings not only support the generally accepted notion that HGT of mutant *ftsI* (complete or partial) contributes to the development of PBP3-mediated resistance in H. influenzae but also strongly indicate that low-rPBP3 strains may acquire second-stage and third-stage PBP3 substitutions through HGT of the SSN-near region as a single recombinational event. This is not surprising, as theoretically, this region may be more easily transferable through transformation than other parts of the *ftsI* gene due to the close proximity to the only complete USS in the gene (nt position 1006 to 1014) (Fig. 5, S3, and S4).

Interestingly, a large number of silent DNA mutations in the SSN-near region of *ftsI*-*2*, *ftsI-4*, and *ftsI*-5 were also present in the older *lambda-2* (Fig. S3 and S4). The *lambda-2* allele, encoding the widespread low-rPBP3 type IIA, was the most frequent resistance-conferring *ftsI* allele in Norway in 2007, present in 30% (35/116) of rPBP3 isolates, belonging to four unrelated STs (24).

10.1128/mSphere.00969-19.3 FIG S3 Alignment of the SSN-near region (nt 1006 to 1295) of the *ftsI* gene in the *ftsI* types represented in the present study (Table 1) and the reference sequence Rd KW20 (53). Text color indicates resistance genotype (Table 1): green, sPBP3; blue, low-rPBP3; red, high-rPBP3. Alignment was performed using Clustal Omega (1.2.4) and visualized using MView (1.63); both tools are available online at https://www.ebi.ac.uk. Amino acid substitutions (positions 350, 357, 377, 385, 389), the uptake signal sequence (USS; amino acids [aa] 336 to 338), and the SSN motif (aa 379 to 381) are indicated. Download FIG S3, PDF file, 0.1 MB. Copyright © 2020 Hegstad et al. 2020 Hegstad et al. This content is distributed under the terms of the Creative Commons Attribution 4.0 International license.

10.1128/mSphere.00969-19.4 FIG S4 Comparison of SNPs (black lines) in the SSN-near region (green shading) of the *ftsI* gene in *ftsI* types lambda-2 (low-level resistance) (24) and *ftsI-2* (high-level resistance) (20). The two types differ in eight positions and possess 19 and 24 SNPs, respectively, of which 17 are shared. Two SNPs are only present in lambda-2, whereas seven SNPs are only present in *ftsI-2*. Red arrows, amino acid substitutions; black arrows, SSN motif; large arrow, uptake signal sequence (USS). Download FIG S4, PDF file, 0.1 MB. Copyright © 2020 Hegstad et al. 2020 Hegstad et al. This content is distributed under the terms of the Creative Commons Attribution 4.0 International license.

We propose that the emergence of high-rPBP3 H. influenzae in Norway in 2006 to 2013 (20) started with the evolution of a distinct high-rPBP3-encoding SSN-near region from the corresponding region of low-rPBP3-encoding *ftsI* alleles belonging to the highly prevalent cluster *lambda* through acquisition of additional point mutations driven by antibiotic pressure. This fragment subsequently disseminated to various low-rPBP3 strains through HGT and transformed them into high-rPBP3 strains. It should be noted that the first high-rPBP3 strains emerged in Norway when the prevalence of low-rPBP3 strains reached 15% (20). This is strikingly similar to the development in Japan 1 decade earlier, when a shift from low-rPBP3 to high-rPBP3 strains occurred when the prevalence of low-rPBP3 strains was approximately 18% (38), suggesting that this level of low-level resistance represents a critical threshold for the development of high-rPBP3 strains in the presence of antibiotic selective pressure.

It should also be noted that strains E and F, carrying the novel ICEs Tn*6685* and Tn*6686*, both possess one of two fourth-stage PBP3 substitutions (Y557) associated with high-level resistance to ESC (cefotaxime MIC, 8 to 16 mg/liter) when they occur concomitantly (25). Including strains E and F, four group III-like+ high-rPBP3 isolates with the Y557H substitution have been reported in Norway (20). Such strains are presumably at particular risk of developing high-level ESC resistance through acquisition of an additional fourth-stage substitution (G555E) (25).

### Conclusion.

The findings in this study illustrate that H. influenzae is capable of acquiring resistance to a wide range of commonly used antibiotics through HGT, in terms of conjugative transfer of ICEs and transformation with chromosomal resistance genes resulting in mosaicism. To our best knowledge, strain G is still the most multidrug-resistant H. influenzae strain reported (20). In addition to the chromosomal and acquired resistance mechanisms present in strain G, transferable genes conferring resistance to macrolides (39), quinolones (40), and aminoglycosides (41), as well as *tet*(M)-mediated resistance to tetracyclines (42), have been reported in H. influenzae. The recently demonstrated potential for further resistance development to critically important beta-lactams such as ESC (25) and carbapenems (43) is a cause for concern. Strains with chromosomally mediated resistance to extended-spectrum cephalosporins, co-trimoxazole, and quinolones combined with MGEs carrying genes mediating resistance to ampicillin, tetracyclines, and chloramphenicol have already been reported, and dissemination of such strains represents a particular worry for the future.

## MATERIALS AND METHODS

### Bacterial isolates.

The included isolates (Table 1) were selected from previous studies (11, 20, 24) based on phenotypic and genotypic characteristics suggesting beta-lactam resistance acquired through HGT of MGE and/or mutant *ftsI* alleles. The strain pairs A/B, C/D, E/F, and H/I consist of genetically unrelated rPBP3 strains sharing identical mutated *ftsI* alleles (nt 1010 to 1719) encoding resistance-conferring PBP3 sequences (20, 24), whereas strain 0 (11) and strains E to H (20) are beta-lactamase positive, indicating horizontal acquisition of a *bla* gene.

### Antimicrobial susceptibility testing.

Table 1 shows phenotypic resistance profiles based on MICs for selected clinically relevant beta-lactam and non-beta-lactam antibiotics, interpreted according to the most recent version of EUCAST clinical breakpoints (44). MICs were determined by broth microdilution (BMD) using custom Sensititre plates (NONAG7; TREK Diagnostic Systems, Thermo Scientific) and MH-F broth according to EUCAST recommendations (45). Strains 0 and A to D were tested at EUCAST Development Laboratory, Växjö, Sweden; strains E to I were tested at Vestfold Hospital Trust.

### Bacterial isolates, DNA extraction, genome sequencing, and assembly.

The isolates were cultured on chocolate agar overnight, and a single colony was transferred to brain heart infusion (BHI) broth and grown overnight in a CO_2_ atmosphere. Genomic DNA was extracted using the Promega Wizard genomic DNA purification kit.

WGS of the study strains was conducted using MiSeq (isolates A to I) or NextSeq 500 (isolates G2, G3, and 0) (Illumina, San Diego, USA). A 2 × 250-bp paired-end library was prepared and sequenced using MiSeq (Illumina) paired-end technology. A library was prepared via the Nextera XT DNA library preparation kit (Illumina) and sequenced using NextSeq 500 and the Mid Output 300 cycles cell. This generated from 2.7 to 8.8 million reads that were trimmed using Trimmomatic (46), resulting in reads ranging from 2.4 to 8.6 million reads. Trimmed reads were assembled with SPAdes 3.10.1 (47). Contigs below 2× coverage and with a length shorter than 200 bp were removed using in-house scripts.

### Phylogenetic grouping of the strains and single nucleotide polymorphism analyses of isolates within strain G.

*In silico* multilocus sequence typing was performed with the MLST tool at the Center for Genomic Epidemiology webpage (48). Individual sequence types were assigned to clonal complexes (CC), defined by the predicted founder, using eBURSTv3 (http://haemophilus.mlst.net/eburst/). Phylogroups were determined by construction of a core genome multialignment by Parsnp (49) using the WGS sequences of our isolates together with those of a reference material (50), reconstruction of a phylogenetic tree by FastTree2 (51), and phylogenetic grouping according to De Chiara et al. (50). Core genome SNPs were called using Snippy 4.3.6 (https://github.com/tseemann/snippy) with strain G as the reference.

### *ftsI*/PBP3 genotyping.

Assignment to PBP3 groups, PBP3 types, and *ftsI* types (Table 1) was performed according to the classification system used in the original publications (20, 24). In short, a 710-bp fragment of the transpeptidase region of *ftsI* (nt 1010 to 1719) was used to define unique *ftsI* types and PBP3 types, whereas PBP3 grouping was based on the presence of amino acid substitutions in positions 385, 389, 517, and 526, as suggested previously (17, 19, 20).

Mapping of *ftsI* SNPs and amino acid substitutions in PBP3 of strains A to I was performed and drawn using the tool Easyfig (52) and later edited with Inkscape 0.92.2. Conserved motifs of particular importance for transpeptidase function (327-STVK, 379-SSN, and 512-KTG) as well as complete 9-bp USS and partial USS with the critical 4-bp core (27) in the reference sequence Rd KW20 (53) were plotted against the sequences of the study strains (Fig. 5 and Fig. S4 in the supplemental material).

In-detail analyses of the SSN-near region were conducted using tools and software available online at http://www.phylogeny.fr (Fig. S2) and https://www.ebi.ac.uk (Fig. S3).

### Bioinformatic analyses to confirm resistance genes and map sequences to MGEs.

Acquired resistance genes were identified using ResFinder with 90% threshold and 60% minimum length (54). Plasmid and ICE structure was found by Basic Local Alignment Search Tool (https://blast.ncbi.nlm.nih.gov/Blast.cgi) and mapping to appropriate sequences. Pairwise comparisons were displayed by Artemis comparison tool (55) using files generated by BLASTn (56). Figures were drawn using Easyfig and edited with Inkscape. ICEs/genomes were annotated with prokka v.1.12 (57).

### Conjugative transfer of ICEs.

A rifampin-resistant spontaneous mutant of H. influenzae Rd (CCUG 18800) made by plating on chocolate agar with 10 mg/liter rifampin was used as recipient strain (Rd-Rif). Filter mating was performed using the following modifications of the method described by Juhas et al. (15). The isolates were grown 48 h on chocolate agar before approximately 10^8^ cells were scraped off the plate and resuspended in 1 ml BHI broth. One hundred microliters of donor cell suspension was gently mixed with 100 μl recipient cell suspension before the mixture was spread at the center of antibiotic-free chocolate plates. Recipient and transconjugants were selected on agar plates containing rifampin (10 mg/liter) for recipients or rifampin (10 mg/liter) plus tetracycline (12 mg/liter) or ampicillin (4 mg/liter) for transconjugants.

### Gap closure, circularization of ICEs, and confirmation of transconjugants.

DNA extraction was performed using NucliSens EasyMAG instrument and reagents (bioMérieux, Marcy-l’Etoile, France) according to the manufacturer’s instructions. JumpStart REDTaq ReadyMix (Merck KGaA, Darmstadt, Germany) was used for PCRs, and BigDye 3.1 technology (Applied Biosystems, Waltham, MA) was used for Sanger sequencing.

Contig gap closure was performed by PCR and Sanger sequencing. A circular form of ICEs was demonstrated by sequencing of PCR products obtained using primers directed outwards from the ends of the ICE (Fig. S1). Transconjugants were confirmed by PCRs specific for the ICE (*bla*_TEM_) and for the recipient (Rd217) (Table 2).

**TABLE 2:** PCR primers used in this study

Circularization ICE	Sequence (5′→3′)		Product size (bp)	Reference
	Forward primer	Reverse primer		
Tn*6685* inverse	GCGTTAGTGGATCGATCGTAG	CACGACGGGTTAAAAACTCA	508	This study
Tn*6686* inverse	CGTAATGTTTGTATCAGCCTTTTT	GTTCTTAAACCGTGGTCAGC	700	This study
Plasmid gap closure				
pH*bla*_TEM_ 1	TAGCTTCCCGGCAACAATTA	AGCGAAATATCTGGGCTGAA	ca. 400	This study
pH*bla*_TEM_ 2	CCCAGATATTTCGCTCTTTCC	TTGTGGGCTGAGTTACAACG	ca. 450	This study
PCRs to confirm transconjugants				
TEM PCR to detect ICE gene *bla*_TEM-1_	ATGAGTATTCAACATTTCCG	CCAATGCTTAATCAGTGAGG	858	58
Rd217 (Rd-ORF specific PCR)	TCTAATTATCGGCGCGATTT	TCACATCACGATGGAAGGAA	463	This study

### Data availability.

The whole-genome sequences of the isolates have been deposited in GenBank under BioProject number PRJNA557131. The novel ICE element sequences have been deposited in GenBank under the accession numbers MN106410 to MN106412.

10.1128/mSphere.00969-19.6 TABLE S2 MICs and clinical MIC breakpoints (mg/liter) used for interpretation (44). Download Table S2, DOCX file, 0.1 MB. Copyright © 2020 Hegstad et al. 2020 Hegstad et al. This content is distributed under the terms of the Creative Commons Attribution 4.0 International license.
