# Supplementary figures and images for: Role of Horizontal Gene Transfer in the Development of Multidrug Resistance in Haemophilus influenzae
Source: mSphere. 2020 Jan 29;5(1):e00969-19. doi: 10.1128/mSphere.00969-19 (PMC6992377; doi:10.1128/mSphere.00969-19)

## Slide 1
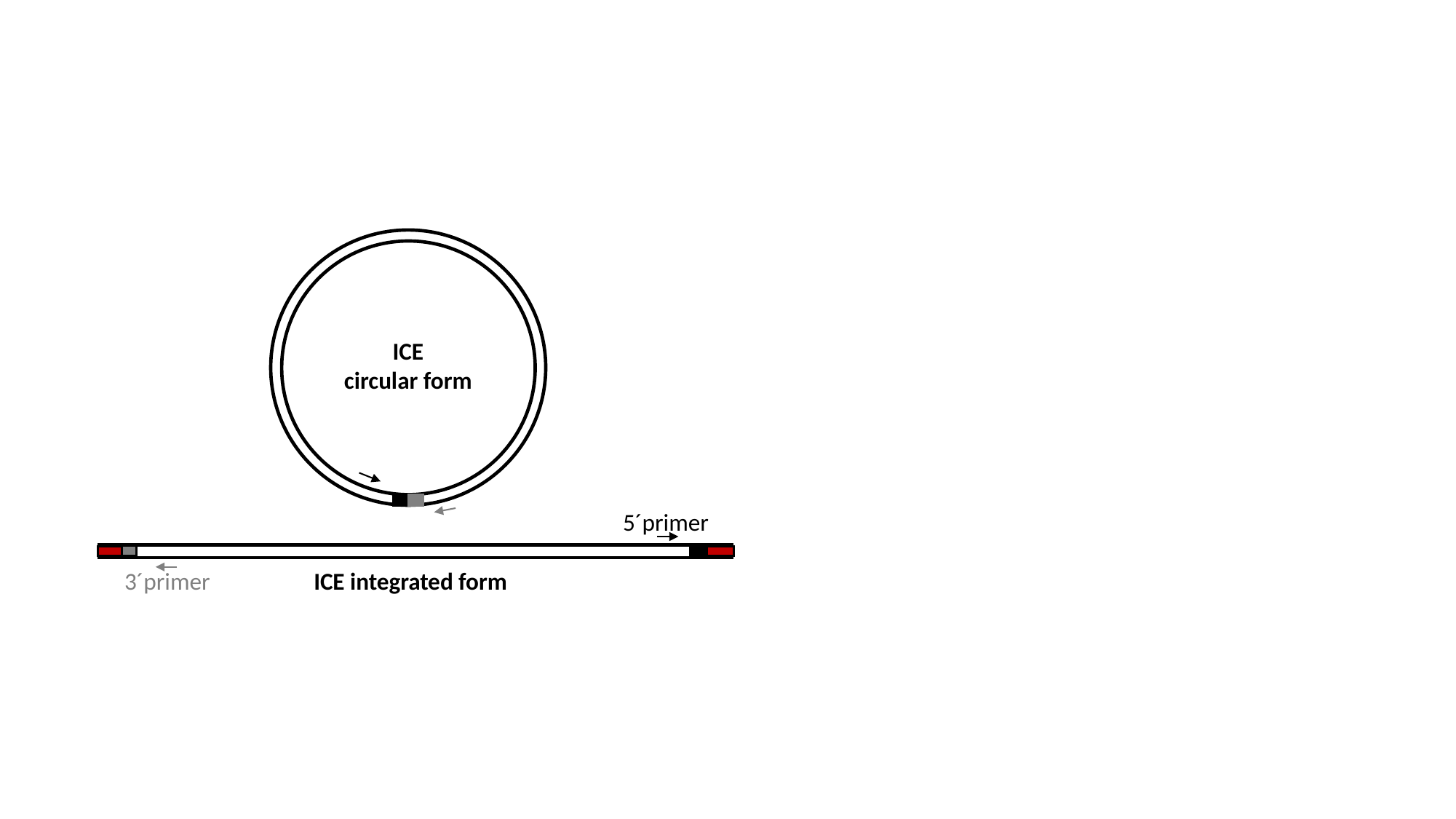

ICE
circular form
5´primer
ICE integrated form
3´primer

Supplement: FIG S1 [file mSphere.00969-19-sf001.pptx]

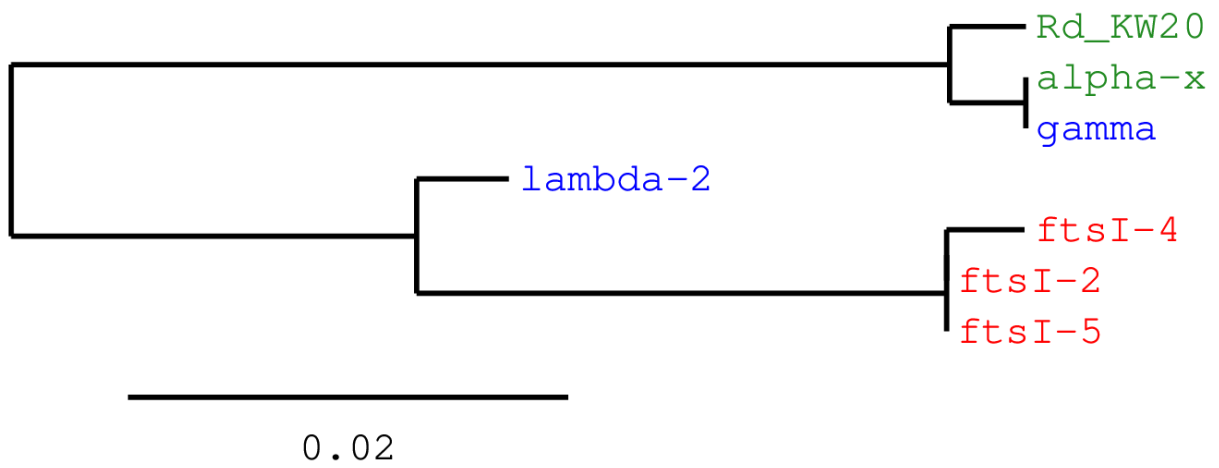

Supplement: FIG S2 [file mSphere.00969-19-sf002.pdf]

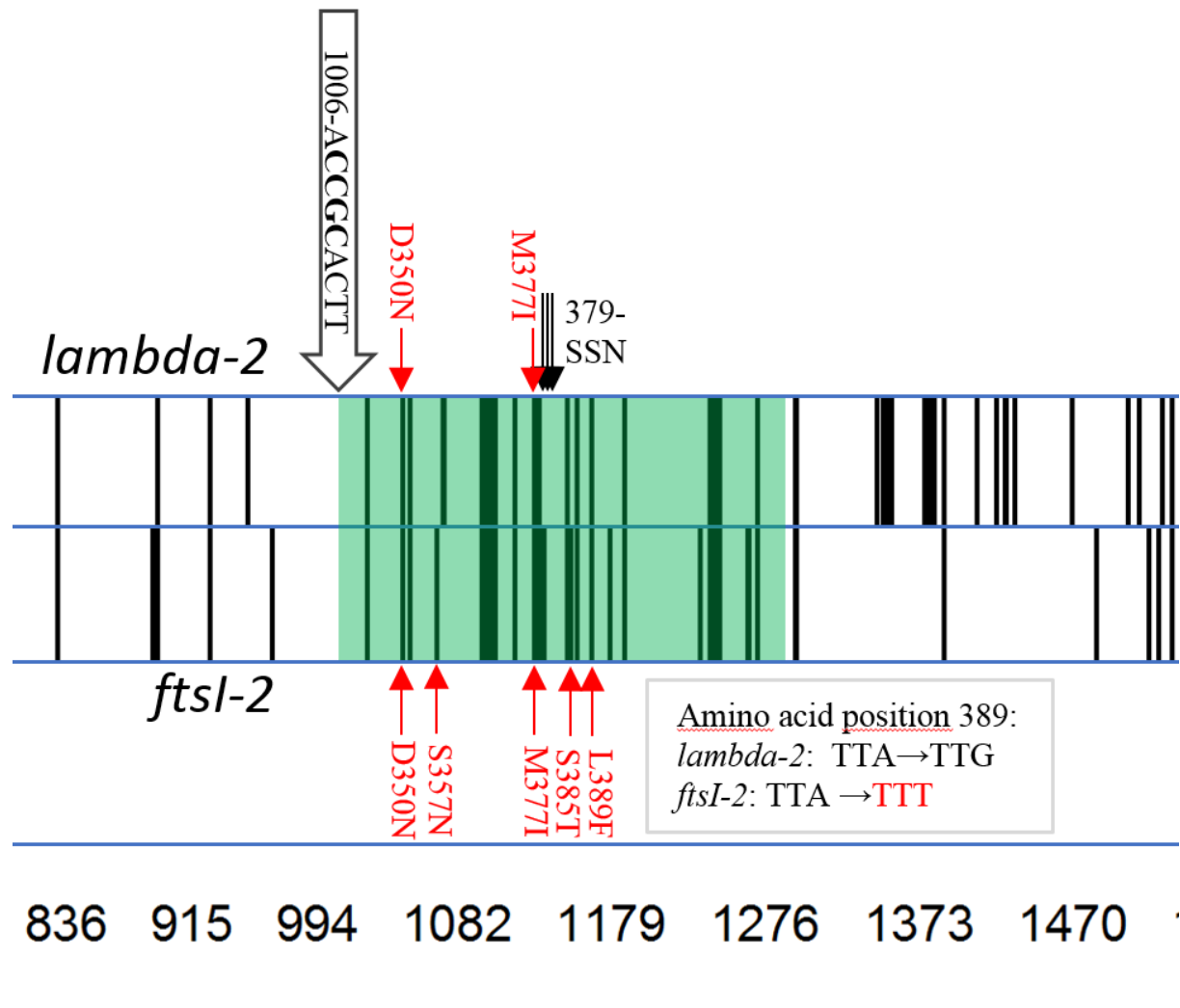

Supplement: FIG S4 [file mSphere.00969-19-sf004.pdf]
