# Supplementary material for: Role of Horizontal Gene Transfer in the Development of Multidrug Resistance in Haemophilus influenzae
Source: mSphere. 2020 Jan 29;5(1):e00969-19. doi: 10.1128/mSphere.00969-19 (PMC6992377; doi:10.1128/mSphere.00969-19)
Supplement: TABLE S1 [file mSphere.00969-19-st001.doc]

|  | **Rd KW20** | ***alpha-x gamma*** | ***lambda-2*** | ***ftsI-2***  ***ftsI-5*** | ***ftsI-4*** |
| --- | --- | --- | --- | --- | --- |
| **Rd KW20** |  | 2 | 19 | 24 | 25 |
| ***alpha-x gamma*** | 2 |  | 18 | 23 | 25 |
| ***lambda-2*** | 19 | 18 |  | 8 | 9 |
| ***ftsI-2***  ***ftsI-5*** | 24 | 23 | 8 |  | 1 |
| ***ftsI-4*** | 25 | 25 | 9 | 1 |  |

Number of SNPs compared to Rd KW20 (53) are indicated. Text color indicate resistance genotype (Table 1): green, sPBP3; blue, low-rPBP3; red, high-rPBP3.
