# Supplementary material for: Role of Horizontal Gene Transfer in the Development of Multidrug Resistance in Haemophilus influenzae
Source: mSphere. 2020 Jan 29;5(1):e00969-19. doi: 10.1128/mSphere.00969-19 (PMC6992377; doi:10.1128/mSphere.00969-19)
Supplement: FIG S3 [file mSphere.00969-19-sf003.pdf]

```
                    ID%    ---USS---                            350                 357
Rd_KW20          100.0%    ACCGCACTTCAACGAGGTGTAGTTAAACGAGATGAAATTATTGATACTACGTCCTTTAAATTAAGCGGTA
alpha-x           99.3%    ACCGCACTTCAACGAGGTGTAGTTAAACGAGATGAAATTATTGATACTACGTCCTTTAAATTAAGCGGTA
gamma             99.3%    ACCGCACTTCAACGAGGTGTAGTTAAACGAGATGAAATTATTGATACTACGTCCTTTAAATTAAGCGGTA
lambda-2          93.4%    ACCGCACTTCAACGAGGTGTCGTTAAACGAGATGAAATTATTAATACCACGTCCTTTAAATTAAGCGGCA
ftsI-2            91.7%    ACCGCACTTCAACGAGGTGTCGTTAAACGAGATGAAATTATTAATACCACGTCCTTTAAATTAAACGGTA
ftsI-5            91.7%    ACCGCACTTCAACGAGGTGTCGTTAAACGAGATGAAATTATTAATACCACGTCCTTTAAATTAAACGGTA
ftsI-4            91.4%    ACCGCACTTCAACGAGGTGTCGTTAAACGAGATGAAATTATTAATACCACGTCCTTTAAATTAAACGGTA

                    ID%                                                          377    ---SSN---
Rd_KW20          100.0%    AAGAAATTGTGGACGTTGCACCACGTGCTCAGCAAACTTTAGACGAGATTTTAATGAACTCTAGTAACCG
alpha-x           99.3%    AAGAAATTGTGGACGTTGCACCACGTGCTCAGCAAACTTTAGACGAGATTTTAATGAACTCTAGTAACCG
gamma             99.3%    AAGAAATTGTGGACGTTGCACCACGTGCTCAGCAAACTTTAGACGAGATTTTAATGAACTCTAGTAACCG
lambda-2          93.4%    AAGAAATTGTGGACGTTGCACCTCGCGCACAACAAACTTTAGATGAGATTTTAATTAATTCTAGTAACCG
ftsI-2            91.7%    AAGAAATTGTGGACGTTGCACCTCGCGCACAACAAACTTTAGATGAGATTTTAATTAATTCAAGTAACCG
ftsI-5            91.7%    AAGAAATTGTGGACGTTGCACCTCGCGCACAACAAACTTTAGATGAGATTTTAATTAATTCAAGTAACCG
ftsI-4            91.4%    AAGAAATTGTGGATGTTGCACCTCGCGCACAACAAACTTTAGATGAGATTTTAATTAATTCAAGTAACCG

                    ID%           385         389
Rd_KW20          100.0%    TGGTGTAAGTCGTCTTGCATTACGTATGCCACCTAGTGCATTAATGGAAACTTATCAAAATGCAGGTTTA
alpha-x           99.3%    TGGTGTAAGTCGTCTTGCATTACGTATGCCACCTAGCGCATTAATGGAAACTTATCAAAATGCAGGTTTA
gamma             99.3%    TGGTGTAAGTCGTCTTGCATTACGTATGCCACCTAGCGCATTAATGGAAACTTATCAAAATGCAGGTTTA
lambda-2          93.4%    TGGTGTGAGTCGCCTTGCATTGCGTATGCCACCTAGTGCATTGATGGAAACTTATCAAAATGCAGGTTTA
ftsI-2            91.7%    TGGTGTGACTCGCCTTGCATTTCGTATGCCACCAAGTGCATTGATGGAAACTTATCAAAATGCAGGTTTA
ftsI-5            91.7%    TGGTGTGACTCGCCTTGCATTTCGTATGCCACCAAGTGCATTGATGGAAACTTATCAAAATGCAGGTTTA
ftsI-4            91.4%    TGGTGTGACTCGCCTTGCATTTCGTATGCCACCAAGTGCATTGATGGAAACTTATCAAAATGCAGGTTTA

                    ID%
Rd_KW20          100.0%    AGTAAACCGACAGATTTAGGCTTGATCGGAGAGCAAGTTGGGATTTTGAATGCAAATCGTAAACGCTGGG
alpha-x           99.3%    AGTAAACCGACAGATTTAGGCTTGATCGGAGAACAAGTTGGGATTTTGAATGCAAATCGTAAACGCTGGG
gamma             99.3%    AGTAAACCGACAGATTTAGGCTTGATCGGAGAACAAGTTGGGATTTTGAATGCAAATCGTAAACGCTGGG
lambda-2          93.4%    AGTAAACCGACAGATTTAGGCTTGATTGGTGAACAAGTTGGGATTTTGAATGCAAACCGTAAACGCTGGG
ftsI-2            91.7%    AGTAAACCGACAGATTTAGGTTTGATTGGTGAACAAGTTGGGATTTTGAACGCAAACCGTAAACGCTGGG
ftsI-5            91.7%    AGTAAACCGACAGATTTAGGTTTGATTGGTGAACAAGTTGGGATTTTGAACGCAAACCGTAAACGCTGGG
ftsI-4            91.4%    AGTAAACCGACAGATTTAGGTTTGATTGGTGAACAAGTTGGGATTTTGAACGCAAACCGTAAACGCTGGG

                    ID%
Rd_KW20          100.0%    CAGATATTGA
alpha-x           99.3%    CAGATATTGA
gamma             99.3%    CAGATATTGA
lambda-2          93.4%    CAGATATTGA
ftsI-2            91.7%    CAGATATTGA
ftsI-5            91.7%    CAGATATTGA
ftsI-4            91.4%    CAGATATTGA
```
