# Supplementary material for: Role of Horizontal Gene Transfer in the Development of Multidrug Resistance in Haemophilus influenzae
Source: mSphere. 2020 Jan 29;5(1):e00969-19. doi: 10.1128/mSphere.00969-19 (PMC6992377; doi:10.1128/mSphere.00969-19)
Supplement: TABLE S2 [file mSphere.00969-19-st002.docx]

| **Strain or isolate** | **Bla** | **A** | **Ac** | **Cf** | **Ct** | **Cx** | **M** | **Tx** | **Te** | **Ch** | **Ci** |
| --- | --- | --- | --- | --- | --- | --- | --- | --- | --- | --- | --- |
|  |  | S≤1  R>1 | S≤2  R>2 | S≤1  R>2 | S≤0.125  R>0.125 | S≤0.125  R>0.125 | S≤2  R>2 | S≤0.5  R>1 | S≤1  R>2 | S≤2  R>2 | S≤0.06  R>0.06 |
| **0** | **+** | >16 | ≤1 | 1 | ≤0.06 | ≤0.06 | ≤0.25 | ≤0.5 | >8 | >8 | ≤0.06 |
| **A** | - | 1 | 2 | 2 | ≤0.06 | ≤0.06 | ≤0.25 | >4 | ≤1 | ≤1 | ≤0.06 |
| **B** | - | 1 | ≤1 | 4 | 0.125 | ≤0.06 | ≤0.25 | ≤0.5 | ≤1 | ≤1 | ≤0.06 |
| **C** | - | 1 | ≤1 | 4 | ≤0.06 | ≤0.06 | ≤0.25 | ≤0.5 | ≤1 | ≤1 | ≤0.06 |
| **D** | - | 1 | 2 | 8 | ≤0.06 | ≤0.06 | ≤0.25 | ≤0.5 | ≤1 | ≤1 | ≤0.06 |
| **E** | + | >16 | 4 | 8 | 1 | 0.25 | ≤0.25 | >4 | ≤1 | ≤1 | 0.12 |
| **F** | + | >16 | 4 | >8 | 2 | 0.5 | ≤0.25 | >4 | >8 | >8 | ≤0.06 |
| **G** | + | >16 | 8 | >8 | 1 | 0.5 | 0.5 | >4 | >8 | >8 | 2 |
| **G2** | + | >16 | 4 | 8 | 1 | 0.5 | 0.5 | >4 | >8 | >8 | 2 |
| **G3** | + | >16 | 8 | 4 | 1 | 0.5 | 0.5 | >4 | >8 | >8 | 2 |
| **H** | + | >16 | 8 | >8 | 1 | 0.25 | ≤0.25 | >4 | ≤1 | ≤1 | ≤0.06 |
| **I** | - | 4 | 4 | >8 | 1 | 0.5 | 0.5 | ≤0.5 | ≤1 | ≤1 | ≤0.06 |

Susceptibility category is indicated with green (susceptible), yellow (susceptible, increased exposure), or red shading (resistant). MICs were obtained with broth microdilution (BMD) according to EUCAST recommendations, using MH-F broth and fixed concentration of clavulanic acid (45). Bla, beta-lactamase; A, ampicillin; Ac, amoxicillin-clavulanic acid; Cf, cefuroxime; Ct, cefotaxime; Cx, ceftriaxone; M, meropenem, Tx, co-trimoxazole; Te, tetracycline; Ch, chloramphenicol; Ci, ciprofloxacin
